# The red and blue light dialog of stomatal regulation for water-efficient crops

**DOI:** 10.1093/plphys/kiag312

**Published:** 2026-05-26

**Authors:** Elena A Pelech, Shellie A Wall, Mae Antonette G Mercado, Tanja A Hofmann, Mengjie Fan, Tracy Lawson

**Affiliations:** Department of Plant Biology, University of Illinois Urbana-Champaign, 286 Morrill Hall 505 S. Goodwin Ave, Urbana, IL 61801, United States; Carl R Woese Institute for Genomic Biology, University of Illinois Urbana-Champaign, 1206 W. Gregory Drive, Urbana, IL 61801, United States; Department of Plant Biology, University of Illinois Urbana-Champaign, 286 Morrill Hall 505 S. Goodwin Ave, Urbana, IL 61801, United States; Carl R Woese Institute for Genomic Biology, University of Illinois Urbana-Champaign, 1206 W. Gregory Drive, Urbana, IL 61801, United States; Carl R Woese Institute for Genomic Biology, University of Illinois Urbana-Champaign, 1206 W. Gregory Drive, Urbana, IL 61801, United States; Department of Plant Biology, University of Illinois Urbana-Champaign, 286 Morrill Hall 505 S. Goodwin Ave, Urbana, IL 61801, United States; School of Life Sciences, University of Essex, Wivenhoe Park, Colchester, Essex CO4 3SQ, United Kingdom; Department of Plant Biology, University of Illinois Urbana-Champaign, 286 Morrill Hall 505 S. Goodwin Ave, Urbana, IL 61801, United States; Carl R Woese Institute for Genomic Biology, University of Illinois Urbana-Champaign, 1206 W. Gregory Drive, Urbana, IL 61801, United States; School of Life Sciences, University of Essex, Wivenhoe Park, Colchester, Essex CO4 3SQ, United Kingdom

## Abstract

Stomatal responses to changing irradiance have significant impacts on photosynthetic carbon gain as well as on plant water use efficiency. These responses can be broken down into the red or mesophyll response which links stomatal conductance with photosynthesis and the specific blue light response that saturates at low fluence levels and is not linked to photosynthesis. Although the 2 pathways are thought to operate independently, there is growing evidence that they are more closely linked than we first appreciated. Understanding both the independence and interplay between red light (RL) and blue light (BL) responses in stomatal behavior could reveal unexploited but critical targets for sustaining or enhancing intrinsic water-use efficiency (*W_i_*) and crop productivity. In this update review, we synthesize the recent advances in our understanding of the RL and BL stomatal responses and propose future research directions to deepen our mechanistic understanding and inform crop improvement strategies.

## Teaser

Recent evidence suggests that the stomatal independent red light photosynthetic response and separate specific blue light response may be more closely linked than first understood.

## Advances box

- The guard cell (GC)-specific blue light (BL) response is species-specific but not universal; the mechanisms responsible for the variation are unknown.
- Red light (RL) and BL stomatal responses are often defined asdistinct pathways; however, there is increasing evidence ofsynergies between them, with the BL component relying on RL.
- Both GC and mesophyll cell photosynthesis is required for activation of the plasma membrane H^+^-ATPase and important for stomatal function.
- Despite its known role in early morning GC opening, BL can have a greater impact later in the diel cycle.
- Emerging technologies and natural variation in BL responses provide opportunities to understand and manipulate BL signal transduction pathways and to breed crops with improved water-use efficiency (*Wi*).

## Outstanding Questions

- What determines species-specific differences in their response to BL?
- What drives diurnal variation in the magnitude of the BL response?
- Can we manipulate the specific BL to improve *W_i_*? If so, how much can we reduce the BL response before other factors impact plant performance?
- How much does GC photosynthesis contribute to stomatal function and to which stimuli?
- What is/are the RL *C_i_*-independent mesophyll signal(s) that coordinate *A* and *g_s_*?
- How are the RL and BL pathways connected and what are the underlying key components?
- If one stomatal pathway was manipulated, would the other compensate?
- What differences are there in stomatal responses to RL and BL between the adaxial and abaxial leaf surfaces?

## Introduction

Stomata, microscopic pores found on most green tissues of higher terrestrial plants, mediate gas exchange between the leaf and the atmosphere, governing CO_2_ uptake for photosynthesis and water loss through transpiration. As such, stomata play a pivotal role in regulating leaf temperature, photosynthetic rates, whole-plant water status, nutrient uptake, and ecosystem-level evapotranspiration (Lawson and Matthews 2020; Pelech et al. 2021; Lawson and Leakey 2024). Stomatal function is often measured and reported as changes in stomatal conductance (*g_s_*), which is the maximum rate of gas exchange (usually H_2_O vapor) between the leaf interior and the atmosphere. Stomatal conductance is determined by anatomical features, including stomatal size and density, as well as functional characteristics such as pore aperture (Lawson and Blatt 2014). Highly specialized guard cells (GCs) surround each stomatal pore whose volume and turgor pressure dynamically regulate stomatal aperture in response to both internal and environmental cues. These changes are brought about through coordinated activity of the plasma membrane H^+^-ATPase, channels and transporters in the plasma membrane of GCs, which are activated via signaling cascades, triggering ion and solute fluxes that adjust the osmotic potential of GCs driving water movement (Kollist et al. 2014; Jezek and Blatt 2017; Lemonnier and Lawson 2024). Environmental factors, including increasing light intensity (particularly red and/or blue wavelengths), increasing air temperature up to an optimum, low atmospheric CO_2_ concentration [CO_2_], and low vapor pressure deficit (VPD), typically promote stomatal opening, while the inverse conditions drive closure (Matthews and Lawson 2019). Internal mesophyll signals and plant hormones are also sensed by GCs to adjust stomatal aperture and optimize *g_s_* (Matthews et al. 2020).

GC responses are central to balancing the carbon demand for photosynthesis with the need to conserve water, making them a novel target for improving water-use efficiency (a measure of plant biomass relative to water use) of our major crops in a warming world (Lawson and Leakey 2024). As may be expected, stomatal behavior and photosynthetic carbon uptake (*A*) are closely correlated (Wong et al. 1979), with increasing aperture removing diffusional constraints on CO_2_ uptake but at the expense of increased water loss. However, stomatal responses are typically an order of magnitude slower than photosynthetic responses to changing environmental parameters (Lawson and Blatt 2014; McAusland et al. 2016; Faralli et al. 2019; Vialet-Chabrand and Lawson 2019), and therefore the relationship between *A* and *g_s_* is often not conserved with short-term noncoordination evident between the 2 parameters (Lawson and Morison 2004; Lawson et al. 2012). In the natural environment, light intensity is one of the most dynamic environmental conditions that not only triggers changes in stomatal aperture but is also a key determinant of photosynthetic carbon uptake rates (Kaiser et al. 2015; Vialet-Chabrand et al. 2016, 2017a, 2017b). Stomatal responses to light can be divided into 2 distinct pathways: the red light (RL) pathway and the blue light (BL) pathway. The RL response, also known as the mesophyll or photosynthetic response, operates at high fluence rates, saturates at light intensities similar to photosynthesis and is slower than the BL response. Photosynthesis in either mesophyll and/or GC chloroplasts is involved in the RL response, with studies using photosynthetic inhibitors consistently reporting the eradication of the RL-induced stomatal opening response (Schwartz and Zeiger 1984). Although, electron transport within GCs has been shown to be as much as 80% of the mesophyll levels (Lawson et al. 2002, 2014) and it is accepted that Calvin cycle enzymes are present, the extent and contribution of GC photosynthesis to stomatal function is an ongoing subject of debate. Early studies reported that as little as 2% of the solute required for stomatal opening was provided by Rubisco activity in *Pisum* (Reckmann et al. 1990); however, as discussed later in this review, GC photosynthetic processes have been shown to contribute to some aspects of stomatal function. In contrast, the BL response is GC-specific and is not inhibited by photosynthetic inhibitors. Typically, the BL response saturates at low fluence rates (∼5 to 20 μmol m^−2^ s^−1^), although saturation is much higher in C_4_ grasses at 120 μmol m^−2^ s^−1^ (Zhen and Bugbee 2020). The BL response is approximately 20 times more effective at stomatal opening than RL and is thought to play an important role in pore opening early in the diel period when the irradiance spectrum is enriched in blue wavelengths to facilitate photosynthetic carbon gain, and/or rapid stomatal responses (Matthews et al. 2020; Vialet-Chabrand et al. 2017a) to sunflecks to maximize photosynthesis (Zeiger and Field 1982). Both the RL and BL response contribute to stomatal opening; however, the magnitude and the extent of each is species-specific (Vialet-Chabrand et al. 2020), which may also play a role in the kinetics of stomatal responses.

While considerable research studies over the last several decades have elucidated the BL-specific signal transduction pathway in GCs and made significant advancements in our understanding of the RL light response, recent studies have identified previously unrecognized molecular and physiological links between these signaling networks that will be the focus of this review. Specifically, shared regulation of the plasma membrane H^+^-ATPase and the dependence of BL responses on mesophyll-driven changes in intercellular CO_2_ concentration *(C_i_*) and metabolic support challenge the long-held view that RL and BL operate independently.

The majority of today's crop canopies are monocultures with high plant density, where leaves experience a high degree of self-shading and transient sunflecking, which is further impacted by the zenith angle of the sun, row orientation, and cloud cover (Long et al. 2022). These dynamics create pronounced fluctuations in irradiance and spectral distribution throughout the crop canopy profile, which impact the diurnal course of stomatal behavior, carbon gain, and ultimately intrinsic water use efficiency (*W_i_*), a measure of *A* relative to water loss through *g_s_* (Vialet-Chabrand et al. 2016, 2017a, 2017b; Matthews et al. 2020). Unraveling the exact mechanisms behind the coordination between *g_s_* and net CO_2_ assimilation (*A_net_*) for the 2 distinct wavelength-dependent pathways of stomatal opening is a fundamental requirement to tackle any canopy-scale challenges to improve *W_i_*. In addition, given that most crops have been domesticated from their wild ancestors adapted to relatively open habitats compared to today's monoculture canopies, variation may exist in the coordination of *g_s_* and *A_net_* and we may have inadvertently selected for differences in RL and BL stomatal responses.

Understanding both the independence and interplay between RL and BL responses in stomatal behavior could reveal unexploited but critical targets for sustaining or enhancing *W_i_* and crop productivity. The available technologies for genetic manipulation, as well as the advancement of transcriptomics and metabolomics technologies can shed light on the effects of stomatal genes in response to red and blue light. In this update review, we synthesize the recent advances in our understanding of the RL and BL stomatal responses, how stomatal behavior and species-specific differences are driven by both red and blue light, diurnal stomatal kinetics under dynamic light environments, and technological advancements. We then propose future research directions to deepen our mechanistic understanding and inform crop improvement strategies.

## Stomatal behavior driven by red light

**Figure 1:**
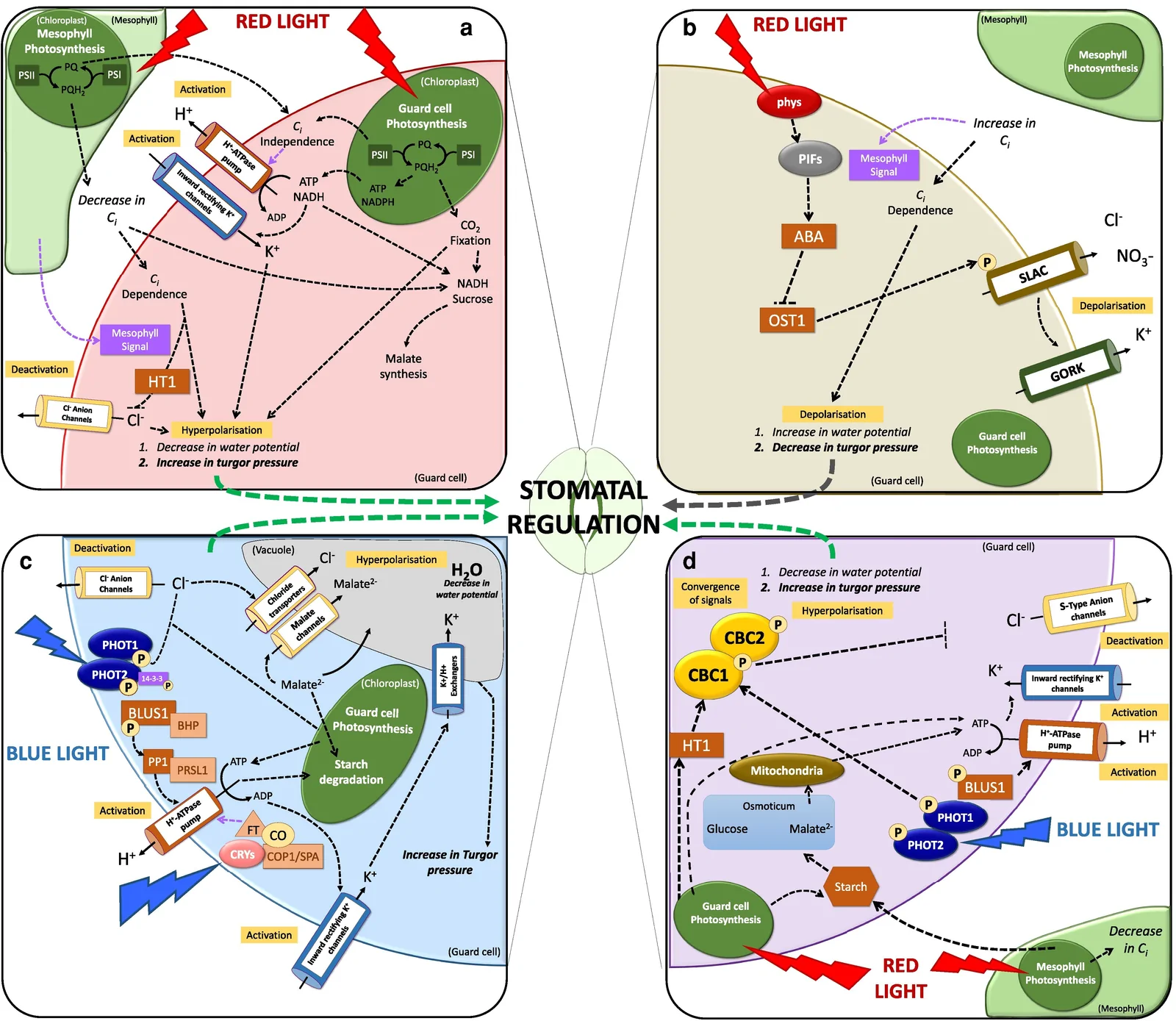
Signaling pathways for stomatal regulation. Signaling transduction pathways include RL-induced opening, which can either begin within guard cell chloroplasts and/or mesophyll chloroplasts (a), RL-induced and darkness induced closing (b), BL-induced opening (c), and both RL- and BL-induced opening (d). Stomatal opening is indicated by the green dashed arrows, and stomatal closing is indicated by the gray dashed arrow. Black dashed arrows represent known signaling cascades, while blunt-ended lines demonstrate signals that suppress ion channel activity. Purple dashed arrows and purple boxes represent hypothetical/unknown signaling cascades and functions. Cells and organelles are indicated within parentheses.

Red wavelengths (620 to 750 nm) initiate RL-induced stomatal opening response (Fig. 1a) which is widely understood to be photosynthesis-dependent and has long been used to explain the tight coupling between *A* and *g_s_* (Wong et al. 1979; Sharkey and Raschke 1981). It has long been assumed that decreases in *C_i_* following mesophyll photosynthesis is the main cue for RL-induced stomatal opening (Roelfsema et al. 2006), and the *A/g_s_* relationship maintains a constant *C_i_*. Research on GCs of albino patches of *Chlorophytum comosum*, which do not contain chloroplasts but still respond to RL, supported the idea that mesophyll-driven changes in *C_i_* drive stomatal opening (Olsen et al. 2002), rather than any direct RL perception in the GCs and there is not a direct role for GC photosynthesis (Roelfsema et al. 2006).

However, several studies reported that stomatal responses to *C_i_* were too low to account for the large light-driven changes in *g_s_* (Wong et al. 1979; Sharkey and Raschke 1981) providing support for an *C_i_*-independent pathway. Several studies have shown that when *C_i_* was kept constant, stomata continued to respond to increasing RL intensity (Messinger et al. 2006; Lawson et al. 2008), while in experiments using the CO_2_-hyposensitive Arabidopsis mutant *ca1ca4*, maintenance of RL-induced stomatal opening was observed (Matrosova et al. 2015; Ando et al. 2022). These findings have led to the hypothesis that *C_i_* is not solely responsible for stomatal opening in response to RL and the coordination between *A* and *g_s_* (see review by Lawson et al. 2018). Several studies have postulated the idea of a direct “mesophyll signal” to GCs (Olsen et al. 2002; Messinger et al. 2006; Wang et al. 2011; Ando and Kinoshita 2018), although the exact nature of this signal remains to be elucidated. Interestingly, a recent study by Zait et al (2025) provides evidence that apoplastic sucrose is the mesophyll signal for regulating GCs ion transport under RL (Zait et al. 2025), which supports earlier studies that suggested an important role of sucrose in stomatal regulations linked to photosynthesis (see Kang et al. 2007; Lawson et al. 2014).

Recent work by Taylor et al. (2024) dissected and quantified the relative contributions of the *C_i_*-dependent RL response and a *C_i_*-independent pathway using wild-type Arabidopsis (Col-0) and CO_2_-hyposensitive mutant *ca1ca4.* Their findings revealed that both mechanisms contributed equally to RL-induced stomatal opening. However, at high *C_i_* concentrations, *C_i_*-dependent closure dominated while *C_i_*-independent opening was suppressed, suggesting molecular crosstalk between the 2 pathways. These authors quantified the contribution of both pathways by measuring *g_s_* response to light when *C_i_* was held constant at various concentrations, which supported the earlier findings (Messinger et al. 2006; Lawson et al. 2008). From these observations it was proposed that the *C_i_*-dependent pathway functions as a rough baseline control of stomatal movements, while the *C_i_*-independent pathway functions as a fine-tuning mechanism of stomatal aperture to enable a more direct relationship with photosynthetic rates (Taylor et al. 2024). Their findings also provide support for an earlier hypothesis that the redox state of the chloroplast plastoquinone (PQ) pool (which reflects the dynamic balance between the light and dark reactions of photosynthesis, integrating excitation pressure at PSII with Calvin cycle activity), serves as a preliminary cue for coordinating *C_i_*-independent stomatal responses. This could also provide the mesophyll photosynthesis-derived signal linking *A* and *gs* (Busch 2014; Kromdijk et al. 2019). Notably, these results complement the work presented by Ando et al. (2022), who concluded that RL-induced phosphorylation of the plasma membrane H^+^-ATPases is a component of the *C_i_*-independent pathway. However, chlorophyll fluorescence measurements used to infer PQ redox state predominantly represent mesophyll chloroplasts (Taylor et al. 2024). Although GC chloroplasts exhibit comparable responses to light and [CO_2_] stimuli (Lawson et al. 2002, 2003), indicating that their redox states may be tightly coupled, their specific role in the signaling pathway remains speculative. The current challenge lies in resolving the cellular origins of each pathway, that is, distinguishing mesophyll-derived signals from those originating within GCs (Fig. 1a). Moreover, much of the current empirical evidence is correlative, complicating efforts to establish causality. Disentangling the molecular regulation behind the *C_i_*-dependent and *C_i_*-independent pathways is thus essential in future research.

Red light photoreceptors phytochromes (phys), in particular phyB and their downstream PHYTOCHROME INTERACTING FACTORS (PIFs) have also been implicated in stomatal light regulation, although current evidence points to a context dependent role rather than a single conserved effect on aperture. In Arabidopsis, overexpression of phyB promoted RL-induced stomatal opening, while pif3 and pif4 mutants display wider stomatal apertures than wild-type, indicating that PIFs negatively regulate light signaling, restraining opening via abscisic acid (ABA)-mediated stomatal closure (Fig. 1b) (Wang et al. 2010). In rice and maize, PIF homologs such as OsPIL15, ZmPIF1, and ZmPIF3 negatively regulate stomatal opening by promoting ABA signaling through activation of key ABA pathway genes, thereby reducing stomatal aperture in response to RL and water-limited conditions (Li et al. 2022). More recent work extends PIF function to diurnal stomatal dynamics in *Arabidopsis*, showing nocturnal accumulation of PIFs which increases expression of the GC K^+^ channel KAT1, required for rapid BL-induced opening at dawn (Rovira et al. 2024). Emerging evidence has highlighted a role of PhyB and PIF4 in light quality integration: low red to far red ratio inhibits stomatal opening in Arabidopsis seedlings through a PIF4 and ABA dependent mechanism, with similar findings observed in cotyledon gas exchange in Chinese kale (Gustavsson et al. 2026). Although these studies together support PhyB as a RL photoreceptor that promotes stomatal opening and PIFs as central transcriptional regulators coordinating stomatal movement with other signals across monocots and eudicots, it is important note that the downstream mechanism and net effect on aperture depend on developmental stage and light environment rather than reflecting a single conserved stomatal module.

## Stomatal behavior driven by blue light

In contrast to the RL-mediated stomatal opening pathway, more research has focused on understanding the signaling pathway of the specific BL response (Fig. 1c) (Shimazaki et al. 2007; Yang et al. 2020; Lemonnier and Lawson 2024; Ivsic et al. 2025). A GC-specific response to BL, independent of mesophyll photosynthesis, was first identified through physiological studies demonstrating that blue wavelengths directly stimulate ion transport and proton extrusion in GCs (Iino et al. 1985; Assmann et al. 1985; Shimazaki et al. 1986), with later molecular studies identifying phototropins as the primary BL receptors mediating this response (Kinoshita and Shimazaki 1999; Kinoshita et al. 2001; Takemiya et al. 2013). This pathway induces a rapid GC-specific response that is effective at operating at relatively low fluence levels to promote stomatal opening, although these levels may be higher than initially reported (Zhen and Bugbee 2020) and species-specific (Vialet-Chabrand et al. 2021). The magnitude of the BL response is also species-specific, with grasses showing a much greater magnitude than dicotyledons, among which there is also considerable variation (Vialet-Chabrand et al. 2021). A recent study has illustrated that the BL response is essential for photosynthetic induction and removing diffusion constraints (Wang et al. 2026), although this was not observed across all species in the study by Vialet-Chabrand et al. (2021), who suggested that *g_s_* was greater than required to remove diffusional constraints. However, the rapid BL stomatal opening may be important for understory species, which rely on carbon fixation during rapid sunflecks (Zeiger and Field 1982).

Blue wavelengths (400 to 500 nm) are perceived by the phototropins (PHOT1 and PHOT2) within the GCs (Shimazaki et al. 1986; Kinoshita and Shimazaki 1999; Briggs and Christie 2002; Doi et al. 2004; Liu and van Iersel 2021; Roeber et al. 2021; Lin et al. 2025), which triggers a signal cascade involving the phosphorylation of BLUE LIGHT SIGNALING 1 (BLUS1). BLUS1 subsequently activates the BLUE LIGHT DEPENDENT H^+^-ATPase PHOSPHORYLATION (BHP) kinase, promoting the phosphorylation activity of the plasma membrane H^+^-ATPase (Kinoshita et al. 2001; Takemiya et al. 2013; Inoue and Kinoshita 2017). The H^+^-ATPase pump utilizes ATP to export H^+^ ions from the GC, leading to rapid hyperpolarization of the plasma membrane, and activation of K^+^ channels that drive stomatal opening via osmoregulation and changes to GC water potential (Assmann et al. 1985; Schroeder et al. 1987; Kinoshita et al. 2001). Cryptochromes (CRYs) also absorb BL (and UV), although there are conflicting reports regarding their role in the BL response (Lascève et al. 1999; Eckert and Kaldenhoff 2000). Experiments in isolated epidermal peels of Arabidopsis stomatal opening was impaired in the *cry1/cry2* mutant (Mao et al. 2005) suggesting a direct role in the GC-specific BL response, and that these photoreceptors function under relatively high BL fluence rates compared with PHOTs (Mao et al. 2005). A direct role for CRYs in BL-induced stomatal opening was questioned by Ohgishi et al. (2004), who explored *phot1-phot2-cry1-cry2* quadruple mutant and triple mutants containing either *cry1* or *cry2* and concluded that the CRYs were not involved in altered pore aperture in response to BL. These conclusions were supported by Boccalandro et al. (2012) who demonstrated that cry1 and cry 2 showed reduced aperture under both BL and RL illumination and suggested that CRYs effects were on ABA levels and were not GC-specific. It has also been suggested that CRYs have an additive effect with phototropins on stomatal opening and that the 2 signals convert on CONSTITUTIVE PHOTOMORPHOGENIC 1 (COP1) (Yang et al. 2017). In Arabidopsis, CRYs interact with CONSTITUTIVE PHOTOMORPHOGENIC 1/SUPPRESSOR OF PHYA-105 (COP1/SPA) complex, leading to the inhibition of light-signaling repressors and the removal of downstream negative regulators (Ando et al. 2013; Cha et al. 2024). A similar COP1-SPA-CRY signaling module has been shown to function in the moss *Physcomitrium patens*, suggesting evolutionary conservation of this BL regulatory mechanism (Kreiss et al. 2023). The resulting accumulation of the transcription factor CONSTANS (CO), which activates transcription of FLOWERING LOCUS T (FT) in the GC, induces stomatal opening via activation of the plasma membrane H^+^-ATPase (Kinoshita et al. 2001; Ivsic et al. 2025). Additionally, COP1 promotes ABA closing and the associated signaling pathway. Recently it has been demonstrated that COP1 regulates GC pH via plasma membrane H ^+^ -ATPase activity (Cha et al. 2024). These studies highlight the complexity of BL-triggered stomatal opening, with the majority of studies focusing on the role of phototropins, while CRY-mediated responses have received much less attention (Fig. 1c). Although the BL response is GC-specific and not directly coupled to mesophyll photosynthesis, it can indirectly influence *A* by mitigating stomatal diffusional limitation. Under steady-state conditions, BL-induced increases in *g_s_* often exceed photosynthetic demand and may reduce intrinsic water use efficiency (Vialet-Chabrand and Lawson 2020; Zhen and Bugbee 2020). In contrast, under fluctuating irradiance such as sunflecks, faster BL mediated stomatal opening can enhance total carbon gain by accelerating *A* induction, with the magnitude of this response differing between species and dependent on environmental conditions (McAusland et al. 2016; Vialet-Chabrand et al. 2017a; Bernardo et al. 2023).

## Stomatal behavior driven by both red and blue light

Stomatal BL responses are greatly influenced by the intensity of RL (Suetsugu et al. 2014) making direct comparisons between studies difficult. To determine the magnitude of the BL response alone, stomatal opening and photosynthesis must be saturated (with RL), as BL will also drive photosynthetic responses, and therefore sufficient RL must be applied in order to maximize mesophyll photosynthesis to determine the specific stomatal BL response. Furthermore, RL saturation is also thought to be needed to provide the ATP required for the BL light response (Suetsugu et al. 2014).

**Figure 2:**
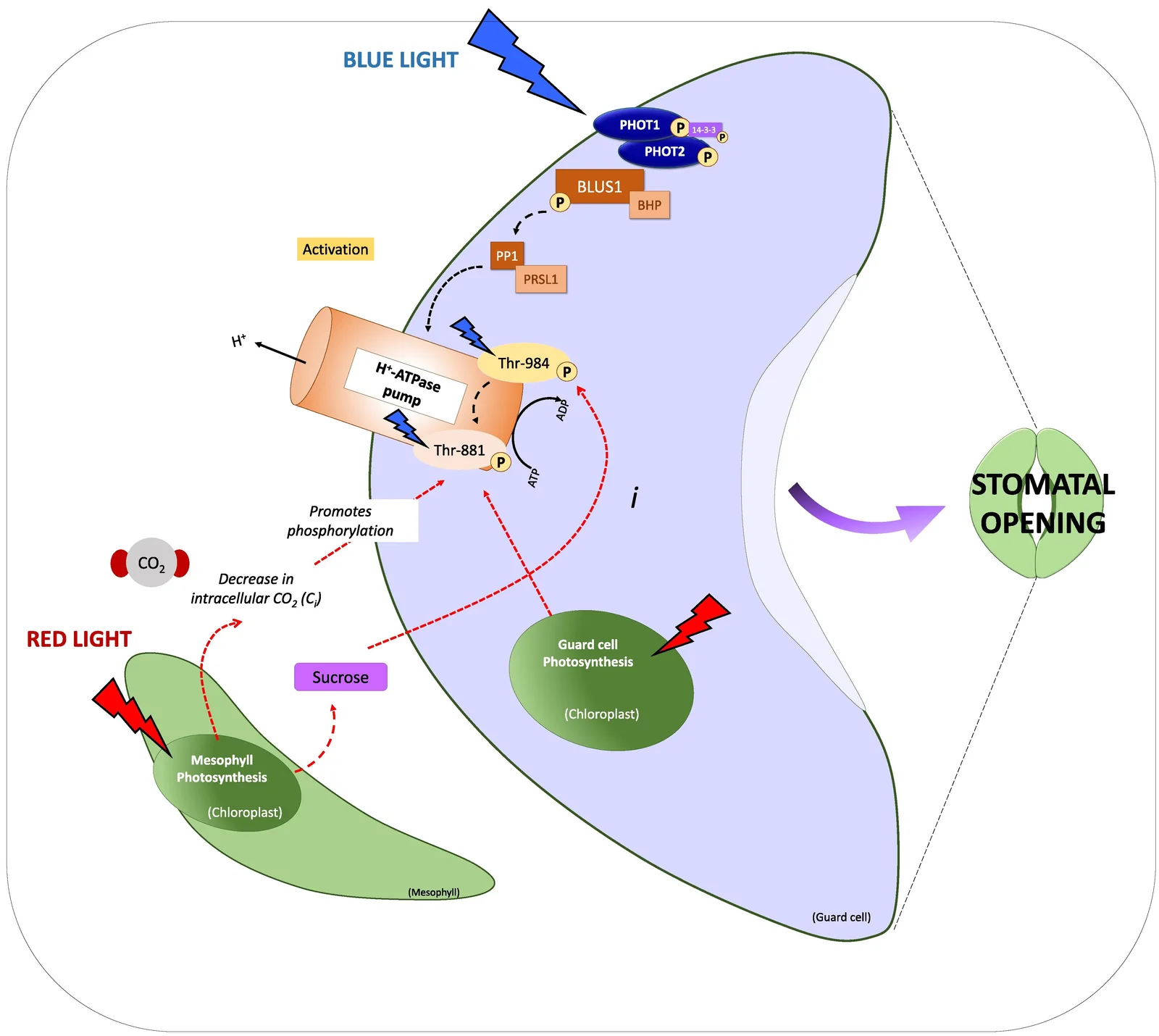
Both red and blue light trigger phosphorylation of the plasma membrane H^+^-ATPase pump. Signaling pathways induced by blue light are indicated by the blue dashed arrows, and those induced by red light are indicated by the red dashed lines. Purple boxes represent hypothetical/unknown signaling cascades and functions. Cells and organelles are indicated within parentheses. Redrawn from Fuji et al. (2024).

Therefore, despite the independence from mesophyll photosynthesis, there is increasing evidence that suggests direct links between the 2 pathways (Fig. 1d and Fig. 2). Two kinases found in the GCs (CBC1 and CBC2 [CONVERGENCE OF BLUE LIGHT AND CO_2_]) link the BL response to low [CO_2_] (and therefore the RL *C_i_*-dependent pathway). Recent studies have established CBC1/2 as central regulators of BL and [CO_2_] signaling, bridging/balancing the demand for both stomatal opening pathways in GCs (Hiyama et al. 2017; Dubeaux et al. 2021; Jones et al. 2022). Positioned downstream of HIGH LEAF TEMPERATURE 1 (HT1) and upstream of SLOW ANION CHANNEL ASSOCIATED 1 (SLAC1), CBC1/2 act as signal mediators of light and [CO_2_] signaling (Takahashi et al. 2025). This position allows them to integrate phototropin-delivered BL signals with *C_i_* (which is associated with the mesophyll RL response) to regulate stomatal aperture according to the most important need of the plant. CBC1/2 contributes by suppressing SLAC1 activity under BL or low [CO_2_] (or low *C_i_*), which limits anion efflux associated with stomatal closure (Fig. 1b). This facilitates hyperpolarization of the GC plasma membrane, membrane H^+^-ATPase activity, which in turn is necessary to drive K^+^ uptake via K^+^-selective channels (Marten et al. 2007; Hiyama et al. 2017; Hayashi et al. 2020; Jones et al. 2022; Takahashi et al. 2025).

The interlink between RL and BL in stomatal opening was further exemplified in work by Hosotani et al. 2021, who demonstrated using constitutive signaling variants of BLUS1 that the BL activation of H^+^-ATPase alone was insufficient for stomatal opening and that a simultaneous reduction of *C_i_* was required. The reduction in *C_i_* directly links the RL *C_i_*-dependent mesophyll photosynthetic pathway with the BL-specific pathway within the GCs. The role of starch degradation in BL-induced stomatal opening (Horrer et al. 2016) also directly links the 2 pathways, as starch in the GCs is thought to be made primarily from sugars imported from the mesophyll and that this uptake may be linked with plasma membrane H^+^-ATPase activity (Flutsch et al. 2020), or from GC photosynthesis (Fuji et al. 2024), once again connecting RL mesophyll photosynthesis with GC requirements for BL-driven responses. While most studies have focused on BL phosphorylation of the plasma membrane H^+^-ATPase in GCs, Ando and Kinoshita 2018 first showed RL-induced activation of this protein in stomatal opening, connecting RL and BL responses.

Progress to unravel the mechanisms underlying the crosstalk between both the BL and RL responses in GCs has recently been made. Using phosphoproteome analysis and site-directed mutagenesis experiments in *Arabidopsis thaliana*, Fuji et al. 2024 and Hayashi et al. 2024 demonstrated that H^+^-ATPase is phosphorylated at several different position during light-induced stomatal opening. Phosphorylation of both Thr-881 and Thr-948 in the C-terminal autoinhibitory domain of the plasma membrane H^+^-ATPase are essential for stomatal opening, and it appears that both RL and BL are involved (Fig. 2). In GC protoplasts of *Vicia faba* and transgenic *Arabidopsis thaliana* phosphorylation of Thr-881 was evidenced under both BL and RL (Hayashi et al. 2024), while Fuji et al. (2024) demonstrated that Thr-948 phosphorylation is a prerequisite for the BL phosphorylation of THR-881. In addition, Fuji et al. (2024) showed that Thr-881 phosphorylation under RL in GC protoplasts was inhibited by DCMU, suggesting that GC photosynthesis is required. However, phosphorylation of Thr-948 by RL did not occur in isolated GC protoplasts and was only observed in intact leaves and suppressed by DCMU. These findings indicate that the involvement of mesophyll cell photosynthesis, (mostly likely through a diffusible signal) is essential for full activation of the plasma membrane H + -ATPase (Okumura et al. 2016). Sucrose has been identified as a mesophyll-derived signal, that enhances RL-induced stomatal opening and links mesophyll photosynthesis with GC ion transport (Fig. 2; Zait et al. 2025). Together these studies clearly indicate that the specific signal transduction pathways first assigned to BL are not BL-specific but can be moderated by RL demonstrating synergy of RL- and BL-induced stomatal opening. A very recent study by Yamauchi et al. (2026) has identified that phosphorylation of WDR48 (directly by PHOTs) is required for starch degradation and BL stomatal opening. This study identifies 2 separate independent pathways for starch degradation involving both WDR48 and BLUS1 and demonstrates that the integration of RL-driven starch synthesis with BL-dependent degradation via these pathways is crucial for stomatal opening in response to red and blue light, providing further support for the coordination between these 2 light signaling pathways.

## Species-specific differences in stomatal responses to red and blue light

Although the core BL signaling pathway is fairly conserved, recent studies reveal clear species-specific differences in the magnitude of the *g_s_* response (Vialet-Chabrand et al. 2021). Early studies established that several fern species within the Leptosporangiopsida (Doi et al. 2006) and selected Polypodiopsida taxa (Doi et al. 2015) lacked a GC-specific BL response, although a subsequent paper has demonstrated a BL response in Polypodiales (Cai et al. 2021). A recent study by McAdam et al. 2025 has shown that stomata in *Equisetum praealtum* and *E. diffusum* respond exclusively to BL, with no RL sensitivity even at high irradiance. A lack of RL response in these plants was postulated to be due to the absence of chlorophyll-containing plastids in *Equisetum* GCs (Cullen and Rudall 2016; Singh and Cullen 2025).

Zhen and Bugbee (2020) examined differences in BL responses in C_3_ and C_4_ species and demonstrated that BL strongly promotes stomatal opening in C_3_ crops (*Helianthus annus* and *Brassica napus*) but plays only a minor role in C_4_ species (*Zea mays* and *Sorghum bicolor*) However, both C_4_ species examined are grasses with dumbbell-shaped stomata and specialized subsidiary cell mechanics, and the species compared belong to different families, making it difficult to attribute these differences solely to photosynthetic pathway. Phylogenetically controlled comparisons that include C_4_ dicots with kidney shaped stomata, such as *Gynandropsis gynandra* and *Flaveria bidentis*, have also reported weaker BL-induced stomatal opening relative to C_3_ relatives (Bernardo et al. 2023), although differences in the RL response were not attributed directly to photosynthesis. It has been suggested that the diminished response in C_4_ dicots was associated with lower GC transcript abundance of the BL photoreceptors PHOT1 and PHOT2, as well as CRY2, in the C_4_  *G. gynandra* compared with C_3_  *T. hassleriana*, proposing that the reduction in phototropin expression accounts for the weaker BL-induced *g_s_* response (Bernardo et al. 2023).

It has been postulated that the GC-specific BL response which results in greater stomatal aperture than required for photosynthesis (Vialet-Chabrand and Lawson 2020) may be important for leaf cooling (Lawson and Matthews 2020), particularly in C_3_ dicots, where photosynthetic carbon gain can be greatly reduced by enzymes kinetics (Rubisco and Rubisco activase) as well as photorespiration (Moore et al. 2021). The carbon concentrating mechanism of C_4_ species suppresses photorespiration and therefore the temperature photosynthetic optimum in these plants is generally much higher than C_3_ species (Sage et al. 2012). Therefore, the lack of BL response in C_4_ dicots may help enhance *W_i_* without the need to regulate leaf temperature (Bernardo et al. 2023). Stomatal BL responses were often thought to be absent in CAM species; however, a recent study on Agave revealed *g_s_* responses to BL, although the effect was only observed in the afternoon and not in the morning. The lack of response in the morning was attributed to high internal CO_2_ concentration, from decarboxylation, which inhibits phosphorylation of HT1 and subsequent activation of CBC1/2 kinase and integrates BL with CO_2_ concentration (Sartori et al. 2025).

A recent study by Bahadar et al. 2025 reported species-specific differences in starch breakdown in response to BL, with no detectable starch degradation observed in *Arabidopsis*, cowpea, and tobacco despite increased stomatal aperture. These findings contrast with the work of Horrer et al. (2016), who demonstrated rapid GC starch degradation within 30 minutes of BL exposure in *Arabidopsis* and showed that this process contributes to efficient stomatal opening by quantifying the starch dynamics directly in GCs using microscopy-based approaches. In contrast, Bahadar et al. (2025) relied on GC-enriched material and bulk starch measurements, which may obscure rapid and cell-specific changes and acknowledged that differences in experimental design, growth conditions, and starch quantification methods may account for the contrasting outcomes. These studies suggest that the contribution of starch degradation to BL-induced stomatal opening is context dependent and may vary according to species, methodology, and experimental conditions. Further GC-specific analysis studies are required to fully elucidate the extent to which starch metabolism contributes to species-specific differences in BL sensitivity and the magnitude of the *g_s_* response.

## Guard cell photosynthesis

The role of GC chloroplasts in stomatal function and in the RL and BL responses has been the subject of intense debate over the last several decades (Lawson 2009; Lawson et al. 2014). Early work on RL-induced stomatal opening using epidermal peels led to the hypothesis that GC photosynthesis supplied sucrose required for osmoregulation and pore opening (Talbott and Zeiger 1993, 1998). Other studies have demonstrated RL accumulation of GC potassium with activation of the plasma membrane H + -ATPase pump (Olsen et al. 2002), with GC photophosphorylation thought to supply the ATP (Tominaga et al. 2001), although RL activation of the H^+^-ATPase pump was not confirmed by Taylor and Assmann 2001. Although the Calvin Benson cycle has been confirmed within GCs, it has also been suggested that GC photosynthesis is too low to support GC osmoregulation (Outlaw 1989; Reckmann et al. 1990). However, GC electron transport has been reported to be 80% of that of the mesophyll (Lawson et al. 2002), and it has been suggested that the Calvin cycle is a major sink for the end products of electron transport (Lawson et al. 2003). More recent studies have suggested that the GC behavior acts more as sink than a source of photosynthate (Santelia and Lawson 2016; Daloso et al. 2017), and studies using transgenic plants with reduced Calvin cycle function in both mesophyll and GCs showed no impact on *g_s_*, which remained the same (von Caemmerer et al. 2004) or even greater than wild-type (Lawson et al. 2008). Therefore, although the role of GC chloroplasts in stomatal behavior remains unclear, they have been shown to be essential for stomatal function (Fig. 1 c, d).

In Arabidopsis mutants in which GC chlorophyll content was reduced, *g_s_* was lower than wild-type (Azoulay-Shemer et al. 2015), and Wang et al. (2014) showed that mutants completely lacking GC chloroplasts had reduced aperture, most likely due to reduced ATP levels. Lower ATP levels in peels compared with intact leaves also suggested the requirement of an additional mesophyll contribution for stomatal function (Wang et al. 2014). A recent study by Lim et al. (2022) used ATP and NADPH sensor proteins to suggest that mitochondria are the main source of ATP and not GC photosynthesis for BL-induced opening (linked to starch degradation), and demonstrated that GC chloroplasts import ATP (generated by mitochondria) from the cytosol via a NUCLEOTIDE TRANSPORTER 1 (NTT1). Mutants lacking NTTs (*ntt1*) had almost no starch in GC chloroplasts and showed impaired stomatal opening. However, isolated GC in wild-type plants illuminated with RL accumulated starch, which was blocked by DCMU, indicating that light-driven photosynthesis electron transport in the GCs supports starch synthesis. Interestingly, isolated GCs of the Arabidopsis *stp1stp4* mutants that lack the plasma membrane monosaccharide-H^+^ symporters SUGAR TRANSPORT PROTEIN 1 and 4, (and therefore lack sugar import capabilities), starch buildup (albeit lower than wild-type) with RL was clearly evident. However, when DCMU was applied, starch accumulation was blocked, clearly concluding that GC photosynthesis provided carbon precursors for starch accumulation and therefore contributes to starch synthesis in GCs.

Zhu et al. (2020) also confirmed earlier studies that GCs in epidermal peels directly respond to RL, which was inhibited by DCMU. As described above, the importance of GC chloroplasts has also been illustrated by Fuji et al. (2024) and was shown to play a direct role in the phosphorylation of specific residues on the plasma membrane H^+^-ATPase to facilitate RL and BL opening (Fig. 2), with mesophyll photosynthesis also playing an important role. All these findings indicate a role for GC chloroplasts in stomatal function, either through the supply of energy in the form of ATP, production of carbon skeletons for osmoregulation, or through sensing and signaling pathways.

## Diurnal stomatal kinetics under fluctuating light with differing red and blue composition

Under natural conditions, irradiance varies in both photon flux and spectrum composition on timescales ranging from seconds to hours, driving non-steady-state *g_s_* that can decouple *g_s_* from *A* (McAusland et al. 2016; Vialet-Chabrand et al. 2017a, 2017b). Spectral composition also changes throughout the day, as scattering and solar elevation alter biologically meaningful blue and red photon ratios (Kotilainen et al. 2020), resulting in the relative contributions of BL and RL pathways to vary across the diel cycle.

**Figure 3:**
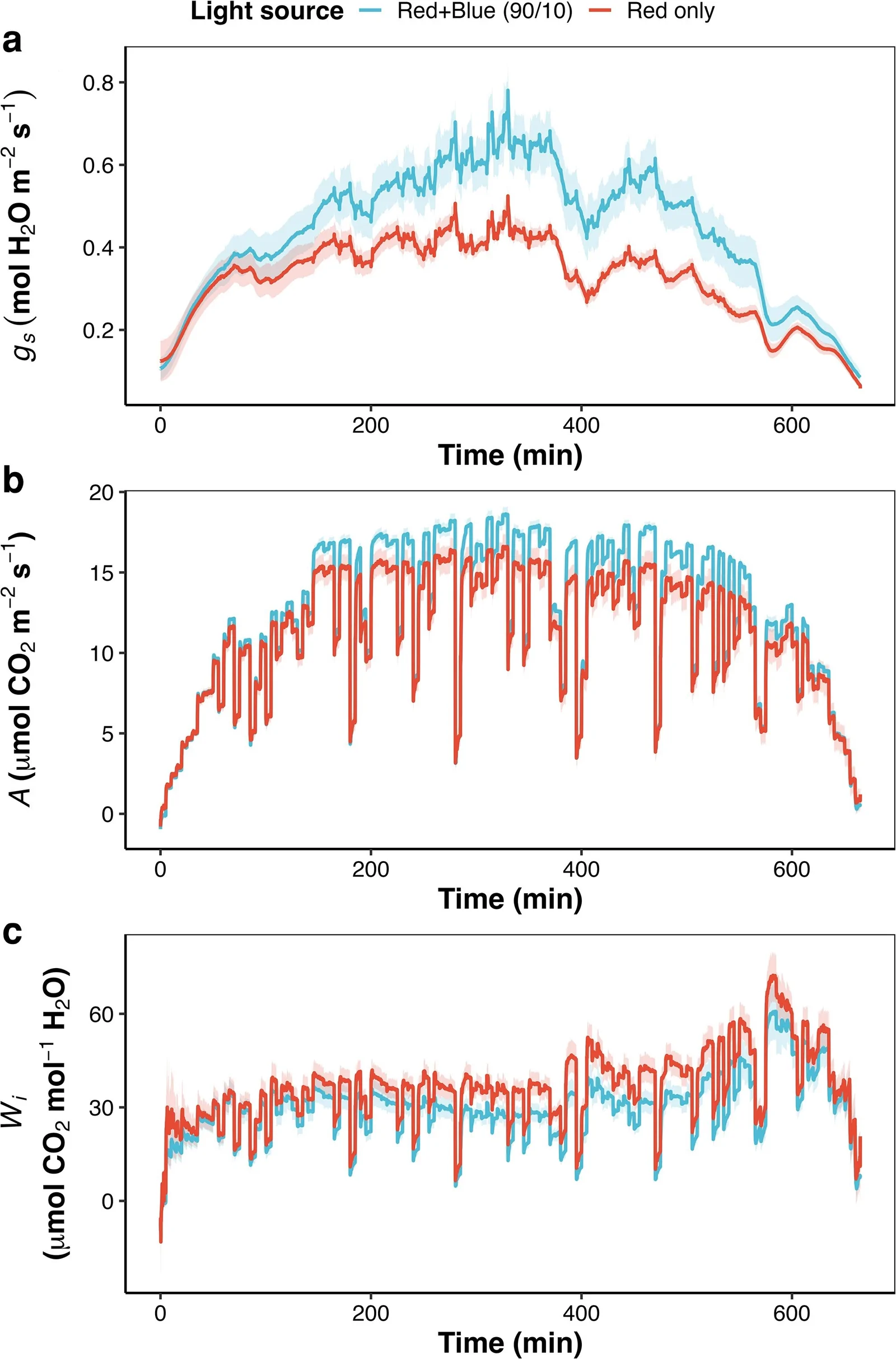
Diurnal illustration of red and blue light effects under stochastic fluctuating irradiance. Time courses of (a) stomatal conductance to water vapor (*g_s_*), (b) net CO_2_ assimilation (*A*), and (c) instantaneous water-use efficiency (*Wi*) measured under a randomized, “natural light” profile with either red + blue (90/10; cyan line) or red only (red line). Mean traces with light shading indicate variability across repeated runs (standard error) (unpublished data of M. Fan & T. Lawson).

In our illustrative dataset (Fig. 3), recorded under a randomized “natural light” profile, a small BL fraction (10%) superimposed on RL (90%) advanced the morning rise in *g_s_* and elevated the daytime plateau. Both treatments also showed a midday depression of *g_s_* and a decline in *g_s_* late in the diel period (Fig. 3a, ∼400 min). *A* tracks light intensity with frequent transient drops as flux decreases; however, *A* plateaus at a higher rate in the presence of RL + BL than RL alone (Fig. 3b). Notably, *A* and *g_s_* do not scale proportionally with the addition of BL, with greater increases in *g_s_* than in *A* reducing instantaneous *Wi* (Fig. 3c). Kinetic asymmetries matter because after a step increase in irradiance, slow stomatal opening transiently constrains *A*, while delayed closure following a decrease in irradiance wastes water and these effects depress diurnal *W_i_* (McAusland et al. 2016; Vialet-Chabrand et al. 2017a, 2017b). A modest BL component increases *g_s,_* diminishing *A* induction losses. However, this greater *g_s_* also raises water loss when stomatal closure lags (Lawson and Blatt 2014; Zhen and Bugbee 2020). BL contributes most to daily *A* when *C_i_* is low (strong RL-driven mesophyll drawdown), and when fluctuations are large and of long duration. Conversely, BL tends to penalize *Wi* when irradiance is lower, and/or fluctuations are of decreasing intensity, or when VPD is high and closure lags prevail.

Blue light effects are largely local to the illuminated surface (Zhen and Bugbee 2020), meaning that adaxial to abaxial patterning and spatial heterogeneity can alter whole-leaf spectral sensitivity across the day. Large area mapping shows that adaxial to abaxial asymmetries and patchiness in stomatal density can change the *g_s_*/*A* covariance that emerges under mixed spectra (Fan et al. 2025b). Notably, reducing surface-specific stomatal density in cereals (ie, wheat) can be compensated by enhanced BL sensitivity, thus preserving performance under RL + BL despite fewer pores (Fan et al. 2025a). Taken together, these observations argue that the “value” of BL is diurnally contextual, as BL is most productive when mesophyll demand is high and diffusional limitation dominates (raising *A* more than transpiration), but it can reduce *Wi* when rapid fluctuations and high VPD make closure delay the limiting factor. Methodologically, this means commonly practiced steady-state step protocols or short induction assays (ie, step changes in light intensity; Vialet-Chabrand et al. (2020) may be insufficient proxies to fully integrate diurnal responses of *A* and *W_i_*. As BL:RL ratio, light dynamics, and plant physiology (circadian phase, hydration) vary over the day, the same plant measured at identical PPFD in morning vs afternoon can show different *g_s_–A–W_i_* relationships. These findings indicate that future studies on the impact of BL on *g_s_* and *A* need validating under realistic fluctuating profiles that include diel changes in spectra (Vialet-Chabrand et al. 2017a, 2017b; Matthews et al. 2019, 2020). This diurnal profile illustrates the significant potential to improve *W_i_* by eliminating or dampening the BL response. As water loss is greater than improvements in carbon gain, minimizing stomatal responses to BL and modifying light-gated channels like GtACR1 (Huang et al. 2021) or XXM2.0 (Huang et al. 2024) could greatly improve *Wi*, with limited impact on *A,* which has potential to improve yield in water-limited environments. However, this approach would be species specific and more appropriate for crops with large stomatal BL responses, such as rice and wheat (Vialet-Chabrand et al. 2021), while crops with a more subtle BL response would be more diffusionally constrained relative to a potential savings in water.

## Advancements in technology to aid understanding of stomatal RL and BL responses

Recent advances in molecular and genomic techniques, such as CRISPR, as well as synthetic biology, have helped uncover the signaling pathways and mechanisms that influence stomatal behavior under BL and RL. However, while these tools have been extensively used, the advancement in spatial transcriptomics and single cells omics will help our understanding of the cascade of regulatory changes in real time in response to various stimuli. As outlined above, advancements in phosphoproteome analysis have proven to be a powerful tool to fully elucidate the intricate details of signal transduction pathways and the phosphorylation targets of key proteins and these tools have been effectively used to demonstrate connections between RL and BL pathways (Hayashi et al. 2024; Fuji et al. 2024). Recently Pullen et al. (2025) described the proteome and phosphoproteome of GC-enriched samples from open and closed stomata, which supported the suggestions that phosphorylation of endomembrane proteins contribute to the regulation of stomatal aperture. Exciting synthetic approaches have also been used to manipulate stomatal kinetic responses to light. Papanatsiou et al. (2019) used a synthetic BL-gated K^+^ channel, BLINK1, to modify ion flux in the GCs and therefore stomatal kinetics, resulting in plants with improved photosynthesis and *W_i_*. These novel approaches demonstrate the potential of using our knowledge of stomatal responses to RL and BL to manipulate these responses for improved crop performance.

While transgenic and -omics approaches provide mechanistic insights, exploring natural variation remains a powerful but underutilized tool to determine species-specific RL and BL responses. There are a number of crop diversity panels that could be used to identify the underlying genes responsible for variation. To date there is no known genome-wide association study examining stomatal RL and BL responses, and this is most likely due to the lack of a high-throughput method of measuring diversity panels. However, exploiting tools such as thermal imaging (Vialet-Chabrand and Lawson 2019, 2020) has the potential to screen large numbers of plants simultaneously for both kinetic responses as well as overall magnitude of *g_s_* changes. Thermal imaging paired with dynamic energy balance modeling has enabled time-resolved maps of *gs* under fluctuating light and wind, providing a quantitative alternative to steady-state thermal indices (Vialet-Chabrand and Lawson 2019, 2020; Fan et al. 2024). Taken together, our advancement in physiological tool development, -omics approaches, and the development of large diversity panels opens up many avenues to discover new targets for crop improvements based on stomatal responses to light.

## Conclusions

Stomatal responses to RL and BL are connected and maybe not as separate as originally perceived, with several studies showing a dependence on RL for the BL response. Although clear differences in the magnitude of the BL response exist between species, the underlying reasons and mechanisms are not clear. Further work is needed to elucidate these, as understanding the mechanisms for this variation has the potential to deliver new targets for potential manipulation to improve *W_i_* (see “Outstanding Questions”). Recent studies have also clearly demonstrated that GC photosynthesis is required for activation of specific residues on the plasma membrane H^+^-ATPase that is required for both BL and RL stomatal opening. These findings reignite questions regarding the role of GC chloroplasts in stomatal function (see “Outstanding Questions”). Understanding both the independence and interplay between RL and BL responses in stomatal behavior could reveal unexploited but critical targets for enhancing *W_i_* and crop productivity.

## Data Availability

The data underlying this article will be shared on reasonable request to the corresponding author.
